# Difference in the periapical status of endodontically treated teeth between the samples of Croatian and Austrian adult patients

**DOI:** 10.3325/cmj.2011.52.672

**Published:** 2011-12

**Authors:** Romana Peršić, Lumnije Kqiku, Gordana Brumini, Medina Husetić, Sonja Pezelj-Ribarić, Ivana Brekalo Pršo, Peter Städtler

**Affiliations:** 1 Department of Endodontics and Restorative Dentistry, University of Rijeka School of Medicine, Rijeka, Croatia; 2 Division of Preventive and Operative Dentistry, Endodontics, Pedodontics, and Minimally Invasive Dentistry, Department of Dentistry and Maxillofacial Surgery, Graz, Austria; 3 IT Department, University of Rijeka School of Medicine, Rijeka, Croatia; 4 Department of Oral Medicine and Periodontology, University of Rijeka School of Medicine, Rijeka, Croatia

## Abstract

**Aim:**

To compare the periapical status of endodontically treated teeth between Austrian and Croatian adult patients and determine its relation to age, sex, position of teeth, and length of root canal filling.

**Methods:**

The study was conducted from 2007 to 2009 at two university dental clinics and included 163 Croatian (412 teeth) and 101 Austrian (430 teeth) patients. We assessed the periapical status of endodontically treated teeth by using the periapical index system and determined its relation to age, sex, position of teeth, and length of root canal filling.

**Results:**

Austrian patients had a greater number of diseased endodontically treated teeth than Croatian patients (*P* = 0.001). In the age group 31-40 years, Austrian patients had apical periodontitis significantly more often (22.1% vs 12.2%, *P* < 0.001) than Croatian patients. In relation to sex and position of teeth, no significant difference was found between the two groups. In Croatian patients, apical periodontitis was significantly more often present in molar than premolar (46.2% vs 29.7%, *P* = 0.022) and front teeth (46.2% vs 24.1%, *P* < 0.001). In Austrian patients, apical periodontitis was significantly more often present in molar and premolar than front teeth (molar-front teeth: 38.2% vs 25.5%, *P* = 0.011; premolar-front teeth: 36.3% vs 25.5%, *P* = 0.029). Croatian and Austrian patients significantly differed in the number of adequately filled and underfilled teeth with AP (both *P*<0.001).

**Conclusion:**

Apical periodontitis was significantly more present in endodontically treated teeth in Austrian patients. The difference in the periapical status between Croatian and Austrian patients was most related to age and length of root canal filling.

Periapical status and root filling quality are frequently assessed to give a complete picture of endodontic diseases and their treatment and predict the treatment outcome. Apical periodontitis (AP) is a chronic inflammatory disorder of periradicular tissues caused by etiological agents of endodontic origin. Persistent apical periodontitis occurs when root canal treatment has not adequately eliminated intraradicular infection (1). It is diagnosed by clinical and radiographic criteria. Since clinical signs and symptoms such as pain, tenderness, swelling, and sinus tract formation occur to varying degrees and are only moderately specific (2), the primary criterion for diagnosis is radiographic interpretation. The most consistent feature of periapical inflammation are bone density changes present in radiographs (3). AP mostly occurs in previously endodontically treated teeth (4), with a greater prevalence in apical than in marginal location (5). The treatment of choice for AP is endodontic treatment or extraction.

During the last decades, there have been several cross-sectional studies of the prevalence of AP (4,6-12). Some epidemiological studies (4,6,10,11,13-18) of treatment outcome have applied the periapical index scoring system (PAI), first described by Orstavik et al (19). This system is attractive since it allows comparisons between the studies (13).

Numerous epidemiological studies, mainly from Scandinavian countries, have investigated the prevalence of AP in relation to an inadequate endodontic treatment (4,14,20,21). Since no epidemiological studies concerning periapical status or root filling quality have been performed on either Croatian or Austrian populations, the aim of the present study was to evaluate the periapical status in endodontically treated teeth in relation to age, sex, position of teeth, and length of root canal filling.

## Methods

### Sample selection

The research was performed at the Dental Clinic of the University of Rijeka School of Medicine, Croatia and the Dental Clinic of the Medical-University of Graz, Austria. It included a convenience sample of 163 Croatian (412 teeth) and 101 Austrian patients (430 teeth) who attended one of the two dental clinics for the first time during the two year period from 2007 to 2009. They sought routine dental care (ie, not emergency care). Only the endodotically treated patients were assessed. Endodontic treatments were performed by their general practitioners. The patients had objective clinical indications to undergo orthopantomography as a diagnostic method with the purpose of further dental treatment planning. The patients who visited the department of Pedodontics and all other patients younger than 18 years were excluded from the study. The patients who visited the department of Periodontology as well as the patients with eight or fewer teeth were excluded because they often had periodontal disease, making it impossible to determine the role played by the endodontic treatment in the occurrence of periapical radiolucencies (9,12,15). The patients who had had endodontic treatment within two-year period were also excluded from the study since it was not possible to conclude whether the periapical lesions were healing or progressing. Since this study was focused on the patients who had visited university dental clinics for the first time, they were considered to reflect the treatment standards of general practitioners.

Data collection for both samples included administering questionnaires (web-extra material) (web extra material 1)and examining orthopantomograms (11,18). All participants agreed to participate in the study by signing an informed consent. They were guaranteed that basic ethical and bioethical principles and personal integrity (independence, righteousness, well-being, and safety) will be respected as regulated by the Nurnberg Codex and the most recent version of the Helsinki Declaration.

### Radiographic evaluation

Orthopantomograms were taken using digital orthopantomograph machines (J. Morita Corporation, Veraviewepocs 6716, Kyoto, Japan and KODAK 8000 digital panoramic system, KODAK Dental System group, Carestream Dental LLC, Atlanta, GA, USA). Images were obtained by compatible software (Mediadent V4, Image Level, Nieuwkerken-Waas, Belgium and Kodak Dental Imaging System, Carestream Dental LLC, Atlanta, Georgia, USA) and laser-printed on film. They were interpreted by two examiners (one in each of the two university departments). The method of viewing the radiographs was standardized – they were examined in a darkened room using a negatoscope and the same magnification (3.5 × ). Both examiners were members of the Restorative Dentistry and Endodontic Department who participated in the pre-doctoral and post-graduate endodontic teaching programs of their Dental Schools.

Orthopantomograms offer advantages over full-mouth periapical radiographs in terms of visibility of a large anatomic region and all teeth on a single radiograph, 10-fold reduction in radiation dose (22), simple and fast taking and processing of radiographs, as well as the possibility to take radiographs in patients who are unable to open their mouth. They also have a high specificity and sensitivity of 86-96% for the detection of periapical pathology. This makes them acceptable for use in epidemiological studies on dental health (23), although it was shown that teeth were better visible in periapical radiographs, with the exception of maxillary second and third molars (3).

Several previous studies used various radiographic parameters to determine the presence of AP (7,24,25). This study assessed periapical status of root-filled teeth using the periapical index scoring system (PAI), proposed by Orstavik et al (19). PAI is based on the use of reference radiographs with verified histological diagnoses (19) and is composed of five categories, each representing a step on an ordinal scale from sound periapical bone to severe AP with exacerbating features. Besides the visual references, two descriptive references, by Kirkevang et al and by Orstavik et al (16,19) were combined and used as follows:

1 – normal periapical structures or normal apical periodontium;

2 – small changes in periapical bone structure or bone structural changes indicating, but not pathognomonic for, apical periodontitis;

3 – changes in periapical bone structure with some mineral loss or bone structural changes with some mineral loss characteristic of apical periodontitis;

4 – demineralization of periapical bone with well-defined radiolucent area or well-defined radiolucency;

5 – demineralization of periapical bone with exacerbating features or radiolucency with radiating expansions of bone structural changes.

The PAI score for multirooted teeth was calculated as the worst score of all roots. When in doubt, higher score was assigned. The threshold between sound and diseased periapical bone was set between the scores 2 and 3 (19). Scores 1 or 2 indicated healthy teeth and scores 3-5 indicated the presence of AP.

The quality of root canal treatment was assessed according to length of root filing. For multirooted teeth, the length of root canal filling was assessed according to the root with the highest PAI score. The length was classified as follows:

1 – adequate – ending 0-2 mm short of the radiographic apex;

2 – underfilling – ending more than 2 mm short of the radiographic apex;

3 – overfilling – ending extruded beyond the radiographic apex.

### Calibration of the examiners

Sixty root-filled teeth (30 from each sample) were taken to provide inter-examiner agreement test considering PAI values and length of the root canal filling. Cohen’s kappa value was 0.898 for PAI scores and 0.928 for length of root canal filling. Intra-examiner agreement for PAI scores of two observers was evaluated by rescoring radiographs of 60 teeth. Kappa values were 0.890 and 0.870, respectively.

### Statistical analysis

Raw data were entered in Excel databases and statistical tests were carried out using Statistica for Windows, release 8.1 (Stasoft, INC., Tulsa, OK, USA). The analyses of the teeth with AP regarding age, sex, position of teeth, and root canal fillings were performed using Pearson χ^2^ test. Post-hoc analysis of the differences within or between groups was performed by *t* test for proportions for independent samples. Chi square test was used to analyze the differences between and within groups. *P* values lower than 0.05 were considered statistically significant. Univariate logistic regression was used to determine the influence of the independent variables: country (Austria/Croatia), age, sex (male/female), position of the tooth (molar/premolar/front), and length of the root canal filling (adequate, underfilling, overfilling) on the dependent variable AP (present/absent). *t* test for proportion difference was used to determine the statistical significance.

## Results

Austrian patients had more root-filled teeth with AP than Croatian patients (χ^2^ = 10.91, *P* = 0.001). There was a significant difference in the number of root-filled teeth with AP between Croatian and Austrian patients according to age (χ^2^ = 11.94, *P* = 0.018; Table 1). In the age group 31-40 years, AP was significantly more present in Austrian than Croatian patients (22.1% vs 12.2%, *P* = 0.007). In other age groups, no differences were found between Croatian and Austrian participants. In Croatian participants, there was a significant difference in the frequency of AP in root-filled teeth according to age (χ^2^ = 31.53, *P* < 0.001; Table 1). AP was significantly more frequent in the age group 41-50 years than in all other age groups (all *P* < 0.05). In Austrian participants, there was a significant difference in the frequency of AP in root-filled teeth according to age (χ^2^ = 29.09, *P* < 0.001; Table 1). In the age groups 31-40, 41-50, and 51-60 years, there were significantly more root-filled teeth with AP than in the age groups under 30 and over 60 years (all *P* < 0.05).

**Table 1:** The difference in the number of root-filled teeth with apical periodontitis (AP) between Austrian and Croatian patients in relation to age

	No (%) of teeth in patients from				
Age (years)	Croatia		Austria		Statistics
	total	with AP	total	with AP	
<30	48	28 (14.4)	36	22 (8.2)	χ^2^ = 11.94 *P* = 0.018*
31-40	64	24 (12.2)	107	59 (22.1)	
41- 50	150	67 (34.4)	114	74 (27.7)	
51- 60	94	45 (23.0)	91	63 (23.6)	
>60	56	31 (16.0)	82	49 (18.4)	
All	412	195 (100.0)	430	267 (100.0)	
Statistics	χ^2^ = 31.53 *P* < 0.001^†^		χ^2^ = 29.09 *P* < 0.001^‡^		

*The number of teeth with AP differs between Croatian and Austrian patients according to age.

†The number of teeth with AP in Croatian patients differs according to age.

‡The number of teeth with AP in Austrian patients differs according to age.

There was no significant difference in the number of root filled-teeth with AP according to sex in either Austrian or Croatian patients. There was also no significant difference between Croatian and Austrian patients (χ^2^ = 1.30, *P* = 0.254; Table 2).

**Table 2:** The difference in the number of root-filled teeth with apical periodontitis (AP) between Austrian and Croatian patients in relation to sex

	No (%) of teeth in patients from				
Sex	Croatia		Austria		Statistics
	total	with AP	total	with AP	
Male	166	88 (45.1)	218	136 (50.9)	χ^2^ = 1.30 *P* = 0.254*
Female	246	107 (54.9)	212	131 (49.1)	
Statistics	*P* = 0.053^†^		*P* = 0.677^‡^		

*No significant differences in periapical status of root-filled teeth according to sex and country.

†No significant differences in teeth with AP of Croatian patients according to sex.

‡No significant differences in teeth with AP of Austrian patients according to sex.

In Croatian participants, there was a significant difference in the number of root-filled teeth with AP according to position of teeth (χ^2^ = 15.35, *P* < 0.001; Table 3.). AP was significantly more present in molar teeth than in premolar (46.2% vs 29.7%; *P* = 0.022) and front teeth (46.2% vs 24.1%; *P* < 0.001). In Austrian participants, there was a significant difference according to the position of root-filled teeth (χ^2^ = 7.57, *P* = 0.023; Table 3). AP was significantly more present in molar and premolar teeth than in front teeth (molar-front teeth: 38.2% vs 25.5%; *P* = 0.011; premolar-front teeth: 36.3% vs 25.5%; *P* = 0.029). There was no significant difference between Austrian and Croatian patients in the number of root-filled teeth with AP in relation to the position of teeth (χ^2^ = 3.44, *P* = 0.179; Table 3).

**Table 3:** The difference in the number of root-filled teeth with apical periodontitis (AP) between Austrian and Croatian patients in relation to position of teeth

	No (%) of teeth in patients from				
Position	Croatia		Austria		Statistics
	total	with AP	total	with AP	
Front	127	47 (24.1)	109	68 (25.5) ^‡^	χ^2^ = 3.44 *P* = 0.179*
Premolars	137	58 (29.7)	147	97 (36.3)	
Molars	148	90 (46.2)^†^	174	102 (38.2)	
Statistics	χ^2^ = 15.35, *P* < 0.001		χ^2^ = 7.57, *P* = 0.023		

*No significant differences between Croatian and Austrian patients in the number of teeth with AP regarding to position of teeth.

^†^Significant differences vs both other positions.

^‡^Significant differences vs both other positions.

In Croatian patients, there was a significant difference in the number of root-filled teeth with AP according to the length of root canal filling (χ^2^ = 117.51, *P* < 0.001; Table 4). The number of overfilled teeth with AP was considerably smaller than that of adequately filled and underfilled teeth (both *P* < 0.001; Table 4). In Austrian patients, there was also a significantly smaller number of overfilled teeth with AP than of adequately filled and underfilled teeth (both *P* < 0.001). The number of overfilled teeth with AP was not significantly higher in Austrian patients (3.7% vs 3.6%; *P* = 0.095). The number of adequately filled teeth with AP was significantly higher in Austrian than Croatian patients (*P* < 0.001; Table 4). The number of underfilled teeth with AP was significantly higher in Croatian patients (*P* < 0.001; Table 4).

**Table 4:** The difference in the number of root-filled teeth with apical periodontitis (AP) between Austrian and Croatian patients in relation to length of root canal filling

	No (%) of teeth in patients from				
Root canal fillings	Croatia		Austria		Statistics
	total	with AP	total	with AP	
Grade I – adequate	188	58 (29.7)	219	127 (47.6)	χ^2^ = 15.42 *P* < 0.001*
Grade II – underfilling	214	130 (66.7)	200	130 (48.7)	
Grade III – overfilling	10	7 (3.6)^†^	11	10 (3.7)^‡^	
Statistics	χ^2^ = 117.51 *P* < 0.001		χ^2^ = 105.24 *P* < 0.001		

*Significant differences in the number of root-filled teeth with AP between Croatian and Austrian patients according to root canal fillings.

^†^Significant differences vs both other root canal fillings.

^‡^Significant differences vs both other root canal fillings.

## Discussion

Austrian participants had significantly more endodonticaly treated teeth with AP than Croatian participants. This can be explained by different standards and indications for tooth extraction in Austrian and Croatian patients. We cannot rule out that many teeth had been extracted because of AP without having considered endodontic treatment or retreatment in previously root-filled teeth.

The design of this cross-sectional study was modified according to other studies (6,16,20,26). According to European Society of Endodontology, for the assessment of periapical condition of endodontically treated teeth it is necessary to have a 4-year observation period (27). We considered a two-year period to be more appropriate, similar to previous research (7,8), since anamnestic information is less reliable if a longer period has elapsed from the endodontic treatment. This is a cross-sectional study, so we did not follow up further status of periapical tissues. A radiograph provides only static information and as the process of periapical healing is dynamic, it is not always possible to conclude whether the periapical lesions are healing or progressing, particularly if less than 2 years has passed from the treatment. Another limitation of cross-sectional studies is that they cannot determine the cause of a disease. We cannot claim that the periapical lesions developed or persisted (not healed) due to persistent intraradicular infection (because of inadequate debridement or filling of root canals, or missed root canals) or because they are true cysts, reactions to extraradicular infection, foreign body reactions, or scar tissue healing reactions. Since usually there is no information available on the history of the missing teeth, it cannot be ruled out that they had been root-filled and then lost to persistent AP (6) or extracted without having considered endodontic treatment. This could explain the greater frequency of endodontically treated teeth with AP in the Austrian than in the Croatian sample. In future studies, clinicians identified by the patients as having performed endodontic treatment and extractions of missing teeth should be contacted to validate the reported data. Furthermore, the patients in this study were those who came to university dental clinics, so the results do not reflect the periapical status in the Austrian or Croatian general population.

AP was present in significantly more molar teeth in Croatian, and premolar and molar teeth in Austrian patients. This can be explained by very complex endodontic anatomy of these teeth. Root canals that are not debrided and filled during endodontic treatment can be a cause of persistent infection of the root canal system, which is the major cause of persistent AP (1). Other variables showed no statistical association with AP.

The success of endodontic therapy depends on three fundamental principles: cleaning, shaping, and obturation of the root canal system. Only the last parameter can be estimated on radiographs so we have no complete information about the quality of the root canal treatment. The density of root filling was not evaluated since it is very difficult to score on orthopantomogram. It was found that incompletely obturated root-filled teeth developed periapical lesions significantly more often than teeth with completely obturated root canals (28). When root canal fillings are within 2 mm of the radiographic apex, the association of endodontic treatment with periapical lesions decreases (29). Root fillings extruded beyond the radiographic apex and fillings limited to the pulp chamber are related to the poorest results (9,21,29). In both Croatian and Austrian patients, the frequency of diseased overfilled teeth was considerably smaller than that of adequately filled and underfilled teeth.

Since some of the major causes of the failure of endodontic treatment are coronal leakage and reinfection of root canal system (10,30-32), in future research we should determine the impact of density of root canal filling and the quality of coronal restoration on periapical health of endodontically treated teeth.
