# Supplementary material for: Difference in the periapical status of endodontically treated teeth between the samples of Croatian and Austrian adult patients
Source: Croat Med J. 2011 Dec;52(6):672–8. doi: 10.3325/cmj.2011.52.672 (PMC3243316; doi:10.3325/cmj.2011.52.672)
Supplement: Supplementary Table 1 [file CroatMedJ_52_s003.pdf]

FDI HEALTH QUESTIONNAIRE

The patient is required to complete the form personally by circling YES or NO. If something is unclear, please ask for clarifications. The data is confidential and shall be used only for medical purposes.

Questionnaire filled out on (date)	Sex: ☐ F ☐ M

Full name	Date of birth	
Profession	Address	Postal code
Phone	Cell	E-mail

Name, address and phone number of closest relative:

PLEASE ANSWER ALL QUESTIONS:

1. Are you suffering from any disease?	NO	YES
2. If yes, which?		
3. Have you been treated over the last two years?	NO	YES
4. If yes, for what?		
5. Full name and phone number of your doctor		
6. Have you been hospitalized over the last two years?	NO	YES
7. Which medical drugs are you taking - occasionally or constantly?		
8. Have you or any member of your family had any complications arising from local or complete anaesthesia?	NO	YES
9. Are you allergic to any drugs and/or anything else?	NO	YES
10. Have you ever experienced problems with blood clotting?	NO	YES
11. Have you ever been exposed to radiation of head or neck?	NO	YES
12. Do you have any infectious diseases?	NO	YES
13. Do you smoke?	NO	YES
If yes, how much?		
14. Are you HIV positive?	NO	YES
15. Are you a drug addict?	NO	YES
If yes, what drug are you using?		
FOR WOMEN		
16. Are you pregnant?	NO	YES
If yes, what is your presumed date of delivery?		

CIRCLE DISEASES (CONDITIONS) YOU HAVE OR HAVE HAD:

☐ Heart valve damage	☐ Endocarditis	☐ Epilepsy	☐ Allergic disorders
☐ Congenital heart defects	☐ Persistent cough	☐ Increased gland size	☐ Viral hepatitis
☐ Bronchiectasis	☐ TBC (tuberculosis)	☐ Gastrointestinal ulcer	☐ Asthma
☐ Thyroid gland diseases	☐ Arthritis	☐ High blood pressure	☐ Leukaemia
☐ Diabetes	☐ Artificial heart valves	☐ Anaemia	☐ Oral candidiasis
☐ Sinusitis	☐ Pacemaker	☐ Glaucoma	☐ Jaundice
☐ Malignant tumour (cancer)	☐ Psychiatric treatment	☐ Venereal diseases	☐ Pulmonary phlegm

Please indicate any other diseases not on this list

SIGNATURE	VERIFIED BY

Please answer all the questions by circling the corect answer.

1.Have You ever visited University dental clinic?

- Yes
- No

2.If yes, which department?

- Departement of Endodontics and Restorative Dentistry
- Departement of Paediatric dentistry and Orthodontics
- Departement of Oral and Maxillofacial Surgery
- Departement of Oral Pathology and Periodontology
- Departement of Prosthodontics

2. Have you ever had?

- Root canal treatment
- Periodontal therapy
- Prosthetic therapy
- Orthodontic therapy
- Tooth extraction, hemisection, root resection or any other surgical procedure

3.Which departement are you currently visiting?

- Departement of Endodontics and Restorative Dentistry
- Departement of Paediatric dentistry and Orthodontics
- Departement of Oral and Maxillofacial Surgery
- Departement of Oral Pathology and Periodontology
- Departement of Prosthodontics

4. Did you have root canal treatment in previous two years?

- Yes
- No

5.If yes, who performed it?

- general practitioner
- specialist in endodontics
